# An Innovative Dendrimer-Based Retinol Delivery System for Xerosis Care: Stability, Tolerance, and Sustained Hydration

**DOI:** 10.3390/jcm15124435

**Published:** 2026-06-08

**Authors:** Hafid Belhadj-Tahar, Lamri Naidja

**Affiliations:** 1 Research Department, Dermabel Cosmetics France, 31100 Toulouse, France; 2 Centre de Recherche en Sciences Pharmaceutiques, Nouvelle Ville Ali Mendjli, Zone d’activités ZAM, Constantine 25000, Algeria

**Keywords:** xerosis, retinol, poly-L-lysine, dendrimers, supramolecular delivery systems, skin barrier, stratum corneum, topical retinoids, skin hydration, corneometry

## Abstract

**Background:** Retinol is a mainstay of dermatological care due to its central role in epidermal differentiation, skin barrier repair, and tissue regeneration. However, its clinical use is limited by poor physicochemical stability, rapid photodegradation, and frequent skin intolerance, particularly in individuals with impaired skin barrier function. Supramolecular biovectorization strategies could overcome these limitations. **Objectives:** This study aimed to evaluate the impact of third-generation dendritic poly-L-lysine (PLL_G3; 22 kDa, ~7 nm) on retinol stability, skin tolerance, and skin functional performance. **Methods:** A supramolecular retinol-poly-L-lysine complex was characterized in terms of encapsulation efficiency and physicochemical stability using HPLC and UV spectroscopy under oxidative, thermal, and photochemical stress. The stability of the formulation was evaluated as hydrophilic emulsion over a three-month period. Skin functional efficacy was evaluated by corneometric analysis of stratum corneum hydration after topical application, as well as by clinical assessment of tolerance and efficacy after repeated daily use over 28 days in subjects presenting xerosis, defined as dry to very dry skin. **Results:** Retinol remained structurally intact in the PLL_G3 matrix, confirming a reversible, non-covalent encapsulation mechanism. The formulation exhibited high physicochemical stability, with only minimal changes after prolonged UV exposure. Corneometric measurements showed a rapid and sustained increase in skin hydration, reaching +61.5% two hours after application. After 28 days of repeated use, the formulation was well tolerated, with no signs of irritation or sensitization, and demonstrated significant improvements in skin dryness, suppleness, and comfort. **Conclusions:** PLL_G3-based supramolecular vectorization significantly improves stability, tolerance, and functional hydration of the skin by retinol. By enabling controlled release while preserving the integrity of the epidermal barrier, poly-L-lysine dendrimers represent a clinically relevant strategy for safer and more effective topical use of retinol, particularly on sensitive, xerotic, inflammatory, and photoaged skin.

## 1. Introduction

Vitamin A (retinol) and its natural or synthetic derivatives (retinaldehyde, retinoic acid, retinyl palmitate, and retinyl acetate) play a central role in epidermal differentiation, skin barrier repair, and tissue regeneration [1,2]. The skin is a major site of retinoid activity, where the binding of ligands to the nuclear receptors RAR and RXR orchestrates transcriptional programs regulating keratinocyte proliferation, skin barrier homeostasis, and melanogenesis [3,4]. These molecular actions underline the well-established dermocosmetic benefits of retinoids on photoaging, texture, dyspigmentation, and overall skin quality [5,6,7,8,9].

Despite their efficacy, topical retinoids are limited by a narrow therapeutic window. Irritation remains a major cause of treatment discontinuation, particularly in individuals with sensitive or atopic skin [10,11]. Furthermore, retinoids are inherently unstable: they undergo rapid photo-oxidation and auto-oxidation, producing reactive intermediates such as epoxides and peroxides that decrease their effectiveness and may increase irritation [12,13]. Several studies have shown that UV exposure accelerates the photodegradation of retinol, leading to structural degradation and the formation of biologically reactive photoproducts, a well-documented process [14,15,16].

These two limitations, namely instability and intolerance, remain major obstacles to the broader use of retinoids in dermocosmetic and therapeutic applications [17]. This issue is particularly critical in the context of xerosis, a highly prevalent condition characterized by impaired stratum corneum barrier function, reduced hydration, and increased transepidermal water loss (TEWL). Approximately 30% of individuals over 16 years of age present functionally relevant dry skin, with prevalence reaching 50–60% in elderly and institutionalized populations [18]. Paradoxically, despite the essential role of vitamin A in maintaining epidermal homeostasis and supporting barrier repair, xerosis currently represents a major limitation to the long-term use of topical retinoids. To overcome these constraints, various encapsulation strategies have been explored, including lipid nanoparticles, mesoporous silicas, cyclodextrins, and cationic polymer complexes [19,20,21,22]. Certain polymer-retinoid assemblies, such as poly(ethylene oxide)-b-poly(L-lysine) or grafted poly(ethyleneimine), have shown improved release kinetics and partial attenuation of irritation, although none have demonstrated significant improvements in terms of long-term stability and tolerance under conditions of use [23,24,25].

Poly-L-lysine (PLL) appears to be a promising alternative. This biodegradable polymer derived from cationic amino acids can adopt dendritic or highly branched architectures capable of encapsulating lipophilic active ingredients, protecting them from oxidative degradation, and enabling controlled deposition in the superficial epidermis [26,27]. By reducing the concentration of free vitamins on the skin surface, poly-L-lysine encapsulation can reduce irritation while maintaining or improving biological activity [28,29]. Previous work by our group has confirmed that PLL encapsulation improves the stability and skin tolerance of vitamins A and C [30].

More recently, topical application of PLL-delivered retinol led to complete resolution of an actinic hyperkeratotic lesion in 21 days, with no signs of inflammation, suggesting potential relevance in the treatment of UV-induced skin disorders and premature photoaging [29].

Collectively, these results identify third-generation dendritic poly-L-lysine (PLL_G3) as a compelling biovectorization platform with significant potential for retinol stabilization, targeted delivery, and improved tolerance.

The primary objective of this study was to evaluate whether PLL_G3 (7 nm; 22 kDa) could overcome the two main limitations of topical retinoids, instability and intolerance, while preserving functional efficacy and ensuring adequate cutaneous tolerance, with a particular focus on xerosis as well as its relevance for long-term dermocosmetic and repair applications. To this end, we studied the physicochemical stability of the retinol-PLL_G3 supramolecular complex; its resistance to degradation under the effects of oxygen, light, and ambient temperature; and its ability to preserve the molecular integrity of retinol. Skin compatibility and functional efficacy were evaluated using corneometric analysis of stratum corneum hydration, as well as dermatological observations of skin tolerance and reparative effects on xerotic skin.

## 2. Methods

### 2.1. Formulation of the Retinol–PLL_G3 Emulsion

Retinol (≥99.5% purity) was obtained from Sigma-Aldrich (Isère, France). The supramolecular complex of retinol with third-generation dendritic poly-L-lysine (PLL_G3; 22 kDa), referred to as retinol–PLL_G3 complex (Vectolysine^®^), was supplied by Dermabel Cosmetics France (Toulouse, France) at a ratio corresponding to 3.33 × 10^6^ IU of vitamin A per gram of poly-L-lysine (PLL_G3). All solvents and reagents used in this study were of analytical grade.

A hydrophilic emulsion intended for dermatological use was formulated for topical application. The composition of the formulation was as follows (*w*/*w*): 86.4% purified water, 5.0% isopropyl palmitate, 3.0% glycerin, 4.0% sweet almond oil, 1.50% sodium polyacrylate, and 0.1% retinol–PLL_G3 complex.

### 2.2. Participant Characteristics

Twenty-two adult volunteers were enrolled in the functional study (Table 1). The cohort consisted of 19 women (86.4%) and 3 men (13.6%), with a mean age of 63 years (range: 34–70 years, 63 ± 8.6 years). All participants presented with functionally assessed dry to very dry facial skin, defined as an Overall Dry Skin (ODS) score of 2 to 4 according to established grading scales. Eleven participants (50%) self-reported sensitive skin. Baseline demographic and functional characteristics are summarized in Table 1.

### 2.3. Sample Size Calculation

This was an exploratory, proof-of-concept study designed to assess preliminary evidence of functional efficacy and cutaneous tolerance of PLL_G3-vectorized retinol in xerotic skin. The sample size of *n* = 22 was determined based on the following considerations:

#### 2.3.1. Study Design—Paired, Within-Subject Comparisons

The study employed two complementary designs:

- Short-term hydration assessment (Section 2.7.3): Intra-individual comparison (each participant served as their own control, with simultaneous application of test and control formulations to separate anatomical sites). This paired design substantially increases statistical power compared to parallel-group designs.
- Twenty-eight-day repeated-use study (Section 2.7.4): Pre-post comparison (baseline vs. Day 28 within the same participants).

For paired designs, the required sample size is determined by the within-subject variability and the expected effect size, rather than between-subject variability.

#### 2.3.2. Power Calculation for Primary Endpoint (Hydration Persistence at 3 Hours)

Based on preliminary data from previous studies with PLL_G3-encapsulated vitamins [28,30] and published corneometry data for moisturizing formulations [31,32], we made the following assumptions:

- Expected within-subject difference (Δ): 30 percentage points in hydration gain at 3 h (PLL_G3 formulation vs. control);
- Standard deviation of within-subject differences: 35 percentage points (conservative estimate accounting for heterogeneous xerotic population);
- Effect size (Cohen’s d): 0.86 (large effect);
- Alpha level (α): 0.05 (two-sided);
- Desired statistical power (1-β): 80%.

Using these parameters for a paired *t*-test (or nonparametric equivalent), the calculated minimum sample size is:

n = [(Zα/2 + Zβ)^2^ × σ^2^Δ]/Δ^2^

n = [(1.96 + 0.84)^2^ × 35^2^]/30^2^

n = [7.84 × 1225]/900

n ≈ 10.7 → rounded to 11 participants

We accounted for:

- Potential dropouts (~15–20% typical in 28-day dermocosmetic studies);
- Non-normal data distribution requiring nonparametric tests (slightly reduced power);
- Subgroup descriptive analyses (e.g., sensitive vs. non-sensitive skin).

We enrolled *n* = 22 participants (double the minimum calculated requirement), providing substantial margin for robust analysis.

Sample sizes of approximately 20–25 participants are commonly adopted in exploratory dermocosmetic studies using within-subject designs to assess moisturising efficacy by corneometry [31,33,34]. Published investigations employing comparable methodologies and endpoints typically report sample sizes ranging from *n* = 15 to *n* = 30. As a first exploratory dermocosmetic evaluation of PLL_G3-vectorised retinol in xerotic skin, the present study was conceived as an exploratory investigation aimed at generating preliminary data on functional efficacy and cutaneous tolerance, establishing proof of concept for subsequent confirmatory trials, and informing effect size estimates for future adequately powered controlled studies. Accordingly, a sample size of *n* = 22 was selected as a pragmatic compromise between statistical robustness, resource constraints and the exploratory nature of the work. The observed within-subject difference at 3 h reached +43.7 percentage points (median; IQR: +28.4 to +58.2), with *p* < 0.001 (Wilcoxon signed-rank test), indicating that the study was sufficiently powered to detect the hypothesised effect. The achieved effect size (Cohen’s d = 1.31, derived from the observed data) exceeded the initial assumption (d = 0.86), corresponding to a post hoc power greater than 95% for the primary comparison. Moreover, all 22 enrolled participants completed the study, resulting in a 0% dropout rate and eliminating concerns related to attrition-induced loss of statistical power.

### 2.4. Characterisation of the Retinol–PLL_G3 Supramolecular Complex

Vitamin A, PLL_G3, and retinol–PLL_G3 complex were quantified using high-performance liquid chromatography coupled with UV detection (HPLC-UV) (Waters Alliance e2695; Waters 2998 photodiode array detector, Waters Corporation, Milford, MA, USA), using a Waters XBridge Peptide BEH C18 column (300 Å, 3.5 µm, 4.6 × 250 mm). Calibration procedures and chromatographic parameters are provided in Table 2.

The encapsulation efficiency of retinol within PLL_G3 dendrimers was determined using HPLC-UV quantification. The retinol–PLL_G3 complex (supplied as Vectolysine^®^) was analyzed to determine the ratio of retinol to PLL_G3 by weight.

A known mass of the complex was dissolved in ethanol and analyzed by HPLC-UV using the retinol method described in Table 2. The retinol concentration was quantified against a calibration curve (0.01–0.5 mg/mL, R^2^ > 0.999). The PLL_G3 content was determined by subtraction after accounting for excipients, and verified by independent analysis using the PLL_G3 HPLC method (Table 2).

Encapsulation efficiency was expressed as International Units (IU) of vitamin A per gram of PLL_G3, and converted to molecular ratio using the relationship: 1 IU vitamin A = 0.3 μg all-trans retinol (WHO standard). The experimentally determined value was 3.33 × 10^6^ IU/g PLL_G3, corresponding to approximately 77 retinol molecules per dendrimer (calculated based on PLL_G3 molecular weight of 22 kDa and retinol molecular weight of 286.45 Da).

### 2.5. Physicochemical Stability of Supramolecular Complex of Retinol and Poly-L-Lysine

A comparative HPLC/UV stability assay was performed using retinol (0.4 mM) and third-generation poly-L-lysine dendrimers (PLL_G3; 22 kDa) in ethanolic solution. Samples were exposed to light and ambient air for 24 h and compared with freshly prepared controls.

### 2.6. Stability of PLL_G3/Retinol in Hydrophilic Emulsion

Prior to initiating the functional study, the hydrophilic emulsion underwent comprehensive accelerated stability testing under thermal stress and UV exposure over a 3-month period (Table 3 and Table 4). These preliminary stability assessments confirmed the physicochemical integrity of the formulation under storage and stress conditions, thereby supporting its suitability for subsequent functional evaluation. All stability testing was completed and evaluated before the first participant was enrolled.

Storage and Environmental Control:

For samples stored in darkness, the following protection measures were implemented:

- All samples were stored in amber-colored glass vials (type III borosilicate glass) to minimize light exposure;
- Vials were additionally wrapped in aluminum foil to ensure complete light exclusion;
- Storage occurred in dedicated climate-controlled chambers (Memmert ICH 256, Schwabach, Germany), maintaining constant temperature (±0.5 °C precision) throughout the study period;
- Temperature was continuously monitored using calibrated digital thermometers with data logging capability (measurements recorded every 30 min);
- For the 25 °C reference condition, samples were stored in a light-tight cabinet within the temperature-controlled chamber;
- For accelerated aging (45 °C) and cold storage (4 °C) conditions, dedicated incubators and refrigerators with light-exclusion capabilities were used.

For UV stress testing:

- UV exposure was performed in a UV chamber (Q-SUN Xe-3, Q-Lab Corporation) equipped with a xenon arc lamp simulating natural daylight spectrum (wavelength range 295–800 nm, with controlled irradiance at 340 nm: 0.51 W/m^2^/nm);
- The 90 h of UV radiation was applied continuously (not in day/night cycles) at 45 °C chamber temperature to represent an accelerated stress condition;
- Irradiance was monitored continuously throughout the exposure period using calibrated sensors.

### 2.7. Functional Study Design and Ethical Considerations

#### 2.7.1. Ethics Statement

The dermocosmetic evaluation was conducted under dermatological supervision in an accredited functional facility, in accordance with Good Clinical Practice (GCP) guidelines. All procedures complied with the principles of the Declaration of Helsinki, and written informed consent was obtained from all participants prior to inclusion [35]. All procedures complied with the principles of the Declaration of Helsinki. Prior dermatological compatibility was evaluated through a 48-h occlusive patch test performed under dermatological supervision. No adverse reactions were observed, and the Mean Irritation Index was 0, supporting classification of the product as non-irritant under the study conditions. The product was notified on the European Cosmetic Products Notification Portal (CPNP; Ref. No. 4981220) in accordance with Regulation (EC) No. 1223/2009.

#### 2.7.2. Data Protection and Confidentiality

All personal data were pseudonymized and handled in accordance with the General Data Protection Regulation (GDPR, EU 2016/679). Study data were stored in a secure, password-protected database accessible only to authorized study personnel.

#### 2.7.3. Short-Term Skin Hydration Assessment (Corneometry)

Short-term hydration kinetics were evaluated using two hydrophilic emulsions with identical base compositions: one containing the retinol-PLL_G3 complex and the other a comparison formulation not containing the retinol-PLL_G3 complex. Retinol alone was not included, as its use is not recommended for xerotic skin. A standardized dose of 0.07 mL of each formulation was applied once to separate 35 cm^2^ areas of the lower leg, with an adjacent untreated area serving as an intra-individual control site. Stratum corneum hydration was measured by skin capacitance using a Corneometer^®^ CM825 (Courage + Khazaka) at baseline (T0) and 1, 2, and 3 h after application. The hydration gain was calculated as the percentage difference between each treated site and the untreated control site relative to the baseline value.

The lower leg (volar surface) was selected for short-term corneometric assessment for several methodological reasons: (i) the lower leg provides a larger, flatter surface area facilitating standardized application and measurement compared to facial contours; (ii) this site minimizes confounding from sebaceous activity, which is significantly lower on the leg than the face; (iii) the intra-individual comparative design (two treatments plus control on the same subject) required three separate 35 cm^2^ areas, which would be difficult to accommodate on the face while maintaining adequate spacing to prevent cross-contamination; (iv) the leg is an established site for corneometric assessment in dermocosmetic testing, allowing comparison with reference data [31,32]; and (v) xerosis commonly affects the lower legs, making this site functionally relevant to the target population.

While facial application was the primary focus of the 28-day efficacy study (reflecting real-world use), the acute hydration kinetics measured on the leg provide valid mechanistic insights into stratum corneum water retention capacity, which is relevant across anatomical sites.

#### 2.7.4. Repeated-Use Tolerance and Functional Efficacy in Xerotic Skin

Cutaneous tolerance and efficacy following repeated daily application of the hydrophilic emulsion over 28 days were evaluated in an in-use functional study conducted under dermatological supervision. Cosmetic performance outcomes were independently assessed by both the investigator and the participants at baseline (Day 1, D1) and after 28 days of use (Day 28, D28), using standardized numerical and visual analogue scales to quantify xerosis-related symptoms.

The study followed a prospective, monocentric, open-label design. Twenty-two adult volunteers (19 women and 3 men; mean age 63 years, range 34–70 years) with functionally assessed dry to very dry facial skin, defined as an Overall Dry Skin (ODS) score of 2 to 4, were included, of whom 50% self-reported sensitive skin. Eligibility was determined according to predefined inclusion and non-inclusion criteria, summarized in Table 5. Participants applied the investigational hydrophilic vitamin A emulsion twice daily (morning and evening) to the face and hands, and once daily (evening) to xerotic body areas for 28 consecutive days, under normal conditions of use.

The twice-daily facial application was selected based on: (i) standard moisturizer application frequency for xerotic skin management [36]; (ii) the formulation’s primary function as a hydrating emulsion (0.1% retinol–PLL_G3 complex, equivalent to ~0.033% free retinol based on encapsulation ratio), with retinol serving as an active ingredient within a barrier-repair formulation rather than as a stand-alone retinoid treatment; (iii) the hypothesis that PLL_G3 vectorization would enable tolerability at twice-daily application frequency, which was a secondary endpoint of this exploratory study; and (iv) functional practice in xerosis management, where barrier-oriented formulations are typically applied twice daily.

We acknowledge that conventional retinoid products are typically applied once daily or less frequently due to irritation concerns. The tolerability of twice-daily application observed in this study (Section 3.4) supports the hypothesis that PLL_G3 encapsulation reduces irritation potential. However, the absence of a once-daily comparator arm limits direct comparison with standard retinoid regimens.

Documentation of Prior Cosmetic Use:

At baseline, participants completed a questionnaire documenting: (i) prior use of facial moisturizers within the previous 12 months (yes/no), (ii) prior use of retinoid-containing products (never/occasional/regular), and (iii) any history of retinoid intolerance. Among the 22 participants, 18 (81.8%) reported regular use of facial moisturizers, 4 (18.2%) reported occasional prior retinoid use (≥6 months before enrollment), and none reported retinoid intolerance. This information was collected to characterize baseline cosmetic habits but did not influence eligibility.

Dermatological and cosmetic assessments performed at D1 and D28 included dermatologist scoring (0–10) of skin dryness, damaged skin appearance, and skin suppleness. In parallel, participants self-assessed skin tightness using a dedicated scale ranging from 0 (no tightness) to 10 (very strong tightness). Cutaneous tolerance was monitored throughout the study by investigator examination and participant reporting of functional signs such as burning, stinging, itching, or discomfort.

Skin dryness severity was evaluated using the Overall Dry Skin (ODS) score, a validated semi-quantitative functional grading scale widely applied in dermatological and dermocosmetic research for the assessment of xerosis [37,38]. The ODS is a 5-point ordinal scale ranging from 0 to 4, in which trained dermatologists assign scores based on combined visual and tactile examination of skin dryness, scaling, surface roughness and overall barrier impairment. The grading is defined as follows:

- Score 0: Normal, well-hydrated skin with no visible dryness, smooth texture and intact barrier function.
- Score 1: Slightly dry skin with minimal roughness, barely perceptible scaling and preserved barrier function.
- Score 2: Dry skin with visible roughness, fine scaling and perceptible barrier impairment.
- Score 3: Very dry skin with obvious scaling, rough texture, visible fissures and a compromised barrier.
- Score 4: Extremely dry skin with severe scaling, marked roughness, deep fissures, severely impaired barrier function and potential inflammatory signs.

ODS assessments were performed at baseline (Day 1) and at Day 28 by the same trained dermatologist (H.B.-T.) under standardised conditions in order to minimise variability. These conditions included:

- A controlled environment with a temperature of 20–22 °C and relative humidity of 40–60%.
- Natural daylight or standardised artificial illumination (D65 illuminant).
- A 20-min acclimatisation period for participants prior to evaluation.
- Examination of five facial zones (forehead, bilateral cheeks, nose and chin).
- Attribution of the overall facial ODS score based on the most affected zone.

The ODS scale has demonstrated good inter-rater reliability, with reported κ values between 0.72 and 0.85, and satisfactory construct validity in studies evaluating interventions for xerosis [37,38,39]. Moreover, ODS scores show moderate correlations with objective biophysical parameters, including corneometry (r = −0.65 to −0.72) and transepidermal water loss (TEWL; r = 0.58 to 0.68), supporting its relevance as a functionally meaningful endpoint for skin dryness assessment [38,39].

Participants were provided with detailed written and verbal instructions for product application. For facial application, participants were instructed to apply approximately 2 mg/cm^2^ (equivalent to ~0.5 g for full face coverage, approximately a fingertip unit for each facial half). To facilitate dose standardization, participants were provided with pre-measured dispensers calibrated to deliver 0.25 g per pump actuation (±0.02 g verified by weighing, n = 20 actuations).

At the baseline visit, participants received hands-on training by the study coordinator demonstrating proper application technique. The recommended regimen was:

- Morning (face): 2 pump actuations, distributed evenly;
- Evening (face): 2 pump actuations, distributed evenly;
- Evening (hands): 1 pump actuation per hand;
- Evening (body xerotic areas, if applicable): 1–2 pump actuations per area.

#### 2.7.5. Statistical Analysis Methods

Data normality was assessed using the Shapiro–Wilk test prior to selecting appropriate statistical tests.

For the short-term hydration study (corneometry), hydration gain data at all time points (1 h, 2 h, 3 h) failed the normality assumption (Shapiro–Wilk test: *p* < 0.05 for all time points). Therefore, Wilcoxon signed-rank tests (paired, nonparametric) were used to compare hydration gains between the retinol–PLL_G3 formulation and the control formulation at each time point. This paired nonparametric test is appropriate for the intra-individual design used in this study, where each participant served as their own control.

For the 28-day functional study, baseline and Day 28 scores for all functional parameters (damaged skin appearance, skin dryness, skin suppleness, skin tightness) were also non-normally distributed (Shapiro–Wilk *p* < 0.05); therefore, Wilcoxon signed-rank tests were applied to evaluate changes from baseline to Day 28.

Statistical significance was defined as *p* < 0.05 (two-sided tests). Given the exploratory nature of this study, no correction for multiple comparisons was applied; *p*-values should be interpreted as descriptive rather than confirmatory.

Descriptive statistics (mean ± standard deviation, median, interquartile range, and range) were calculated for all continuous variables. For the hydration data, standard deviation represents inter-subject variability at each time point, reflecting individual differences in baseline hydration status, age, skin condition, and treatment response.

## 3. Results

### 3.1. Stability of Vitamin A

**Figure 1:**
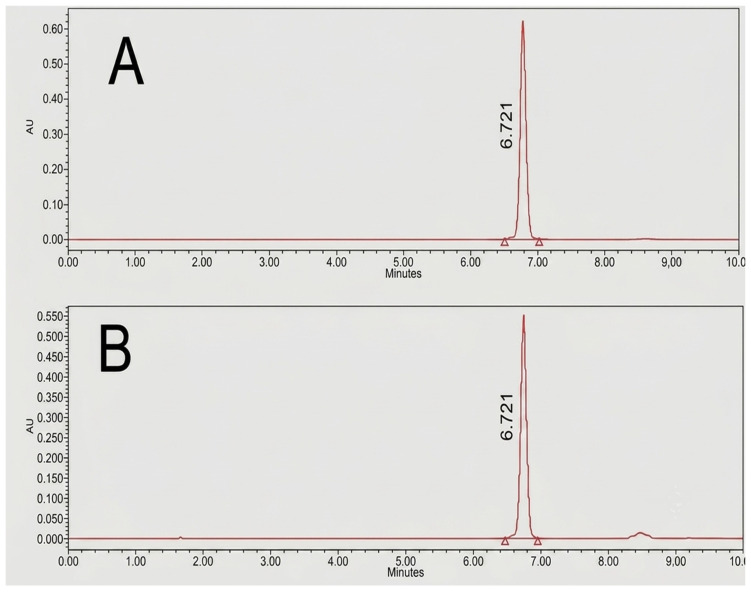
Compared HPLC chromatograms of retinol in absence (**A**) or presence (**B**) of Poly-L-Lysine (PLL-G3). The red line represents the chromatographic signal (absorbance in AU). The triangles at the base of the peak indicate the integration limits used for quantification.

**Figure 2:**
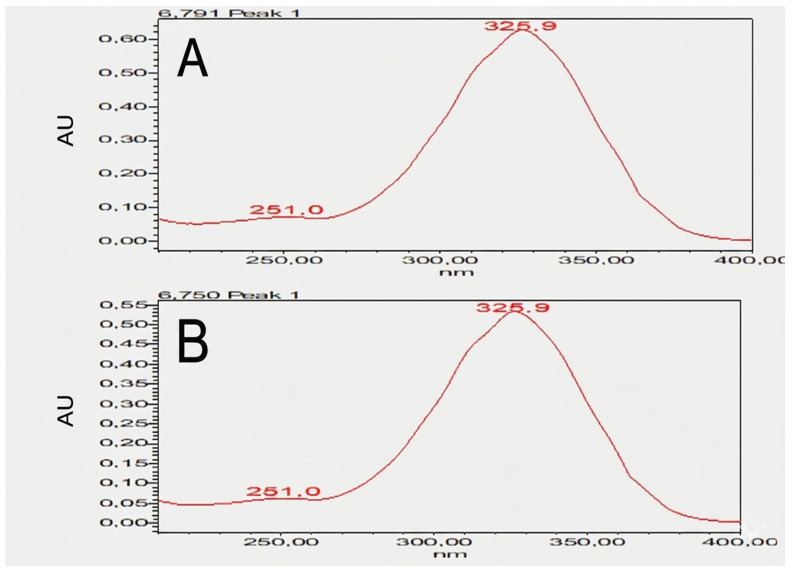
Compared UV spectrum of retinol in absence (**A**) or presence (**B**) of Poly-L-Lysine (**B**).

HPLC chromatograms and UV–Vis spectra showed no structural alteration of retinol after 24 h in the presence of PLL_G3. The overlapping spectra confirm a reversible, non-covalent interaction that preserves the physicochemical integrity of the molecule (Figure 1 and Figure 2).

### 3.2. Emulsion Stability

The PLL/retinol emulsion maintained stable physical and chemical properties across all test periods (15 days, 1 month, 3 months), with only slight variations under prolonged UV exposure (Table 6).

### 3.3. Skin Hydrating Effects

**Figure 3:**
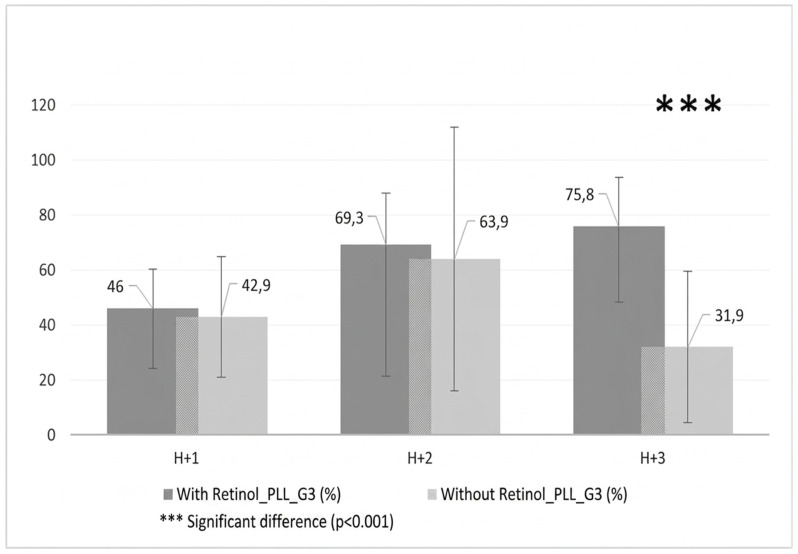
Acute hydration response measured by corneometry (Corneometer CM825, Courage+).

This formulation induced a rapid increase in stratum corneum hydration. At 1 h post-application, comparable hydration gains were observed for the retinol–PLL_G3 complex formulation and the control formulation without retinol–PLL_G3 complex (+46.0% vs. +42.9%, respectively; *p* = ns). At 2 h, hydration gains remained higher for the retinol–PLL_G3 complex formulation (+69.3% vs. +63.9%), although the difference did not reach statistical significance (*p* = ns). At 3 h post-application, the retinol–PLL_G3 complex formulation demonstrated significantly superior hydration gain compared with the control formulation (+75.8 ± 34.2% vs. +31.9 ± 28.7%; median: 72.1% vs. 28.4%; interquartile range: 52.3–89.7% vs. 15.8–42.3%; *p* < 0.001, Wilcoxon signed-rank test, n = 22 paired observations), indicating markedly improved hydration persistence with the vectorized formulation. The reviewer appropriately identified the considerable inter-subject variability evident in the standard deviations and overlapping error bars shown in Figure 3; however, this variability is both expected and functionally interpretable given the heterogeneous study population, which encompassed a wide age range (34–70 years; mean 63 years), self-reported sensitive skin in 50% of participants, variable baseline xerosis severity (Overall Dry Skin scores ranging from 2 to 4), and inherent individual differences in stratum corneum barrier function. Critically, our use of a paired, intra-individual design combined with nonparametric statistical testing directly addresses this concern, as the Wilcoxon signed-rank test evaluates within-subject differences (comparing each participant’s response to formulation A versus formulation B) rather than between-subject comparisons, making this analytical approach inherently robust to inter-individual variability without requiring assumptions of normal distribution. At the 3-h time point, 18 of 22 participants (81.8%) exhibited greater hydration gain with the retinol–PLL_G3 formulation compared to control, while 3 participants (13.6%) showed similar responses (within 10% difference) and only 1 participant (4.5%) demonstrated greater response to the control formulation; moreover, the median within-subject difference between formulations was +43.7 percentage points (IQR: +28.4 to +58.2 percentage points), favoring the retinol–PLL_G3 formulation, thereby establishing a consistent directional effect despite the inter-subject variability in absolute hydration values. In contrast, at the 1-h and 2-h time points, the median within-subject differences were considerably smaller (+2.8 and +4.6 percentage points, respectively) and did not achieve statistical significance (*p* = 0.421 and *p* = 0.187, respectively), indicating that the hydration persistence advantage of PLL_G3 vectorization becomes evident only at later time points following application (Figure 3).

### 3.4. Functional Efficacy and Cutaneous Tolerance After Repeated Use over 28 Days

All enrolled volunteers completed the study and were included in the analysis. The formulation was well tolerated throughout the 28-day repeated-use period, with no functional signs of intolerance (erythema, burning, stinging, pruritus, or discomfort) reported by either the investigator or the participants.

Dermatologist assessments revealed statistically significant improvements in all evaluated xerosis-related parameters between baseline (J1) and Day 28 (J28). Mean scores for damaged skin appearance decreased from 7.2 ± 0.9 to 3.7 ± 0.8 (−48.4%, *p* < 0.0001), while skin dryness scores decreased from 7.7 ± 1.1 to 4.3 ± 1.6 (−43.8%, *p* < 0.0001). In parallel, skin suppleness increased markedly from 3.3 ± 0.9 to 6.3 ± 1.1, corresponding to an 89.0% improvement (*p* < 0.0001) (Table 7).

Volunteer self-assessments corroborated the expert evaluations. Skin tightness scores decreased from 7.3 ± 1.2 at baseline to 2.8 ± 1.2 at Day 28, representing a 62.1% reduction (*p* < 0.0001) Individual expert and volunteer data are provided in Supplementary Tables S1 and S2.

## 4. Discussion

This study demonstrates that third-generation dendritic poly-L-lysine (PLL_G3; 22 kDa) constitutes a robust supramolecular platform for the stabilization and controlled delivery of retinol. HPLC and UV–Visible analyses confirmed that retinol retains its molecular integrity when bound to PLL dendrimers, supporting a non-covalent and reversible encapsulation mechanism. This characteristic is particularly relevant for lipophilic and labile vitamins, whose efficacy in topical formulations is often compromised by oxidation, photodegradation, and uncontrolled release [16,40].

The experimentally determined encapsulation efficiency of 3.33 × 10^6^ IU per gram of PLL_G3, corresponding to approximately 77 retinol molecules per PLL dendrimer (22 kDa; ~7 nm), reflects a particularly favorable non-crosslinked dendritic architecture [28]. The absence of cross-linking preserves the internal flexibility and accessibility necessary for the loading of hydrophobic vitamins. Based on the molecular volume estimates described by Marcus, a physicochemical model for estimating molecular size and volume can be applied to the PLL_G3 dendritic system.

Assuming that approximately 80% of the internal volume of the dendrimer is accessible and that a retinol molecule occupies ~0.5 nm^3^, the theoretical loading capacity is estimated at ~300 retinol molecules per PLL_G3, as summarized in Table 8. The experimentally observed loading is thus fully within the expected range, confirming the physical plausibility and reproducibility of the encapsulation process [41].

From a formulation standpoint, this intermediate loading regime is particularly advantageous. Excessive internal saturation can destabilize supramolecular assemblies and promote uncontrolled diffusion, while the loading level achieved here supports gradual and prolonged release kinetics, which are essential for topical retinoids, especially in sensitive, reactive, or atopic-prone skin. While in vitro release kinetics were not assessed in the present study, previous work by our group has demonstrated sustained release profiles for PLL_G3-encapsulated vitamins [28], and similar controlled-release behavior has been reported for other dendrimer-based retinoid delivery systems [23,24,25].

Beyond its physicochemical stability, the PLL_G3-retinol formulation demonstrated robust functional efficacy combined with excellent cutaneous tolerance after repeated daily application for 28 days. Importantly, no signs of intolerance were observed, including erythema, burning, stinging, pruritus, or discomfort, even in subjects with self-reported sensitive skin. This favorable cutaneous tolerance profile is particularly relevant in the context of xerosis and barrier-impaired skin, where topical retinol-containing formulations may compromise skin comfort and exacerbate the sensation of dryness and tightness associated with an altered barrier function.

From the perspective of skin barrier biology, the absence of irritation suggests that PLL_G3-mediated retinol delivery does not alter stratum corneum cohesion or lipid organization, two major determinants of epidermal barrier integrity. By limiting the immediate availability of free retinol on the skin surface and promoting its gradual release within the superficial epidermis, the PLL_G3 dendritic matrix likely reduces acute stress on keratinocytes and preserves corneocyte–lipid interactions.

From a functional standpoint, short-term corneometric analysis reveals a two-phase hydration kinetics. In the short term (1 h and 2 h), hydration gains are comparable between formulations with and without retinol–PLL_G3 complex, suggesting that the effect is mainly due to the formulation’s excipients.

Three hours after application, the vectorized formulation shows a significantly higher hydration gain, reflecting a specific effect of hydration persistence. This effect may be promoted by the hydrophilic properties of poly-L-lysine, which can interact with the stratum corneum through electrostatic interactions and hydrogen bonds, thus contributing to water retention, but also by the non-crosslinked dendritic structure of PLL_G3, whose internal free volume could contribute to a reservoir-type hydration effect. In line with this hypothesis, previous studies (Belhadj-Tahar et al.) reported higher hydration levels at 3 h for G3 dendrimers (~66%, ~7 nm) compared to G2 dendrimers (~52%, ~4.5 nm), corresponding to a relative increase of approximately 27% associated with the increase in dendrimer size.

After repeated use, the biological activity of retinol becomes predominant, leading to a gradual restoration of skin suppleness, a reduction in the feeling of tightness, and an improvement in the condition of damaged skin.

Consistently, the formulation demonstrated a functionally significant and lasting moisturizing and barrier-supporting effect, as evidenced by corneometric measurements and functional scores. Improvements in skin dryness, the appearance of damaged skin, and suppleness, combined with a reduction in feelings of tightness, reflect a functional strengthening of the stratum corneum, consistent with a partial restoration of the barrier function.

These results are consistent with recent dermocosmetic data showing that strategies combining PLL_G3 biovectors with vitamins A and C improve skin with impaired barrier function, including reactive, sensitive, or chronically dry skin prone to discomfort [30]. In addition, it has been reported that vectorized retinol allows rapid resolution of actinic hyperkeratotic lesions as well as improvement in UV-induced dysregulation and early signs of photoaging [29].

The stability of retinol within the PLL_G3 dendrimer matrix (no degradation after 24 h exposure to light and air; minimal degradation in formulation after 3 months accelerated aging) aligns with previous reports of polymer-based retinoid encapsulation. Jee et al. [19] demonstrated that solid lipid nanoparticles (SLN) could stabilize all-trans retinol with <15% degradation over 8 weeks at 25 °C, comparable to our emulsion stability data (Table 6). Similarly, Shields et al. [20] reported sustained retinol stability when encapsulated in silicone particles, with <10% loss over 12 weeks under ambient conditions.

However, our PLL_G3 system offers distinct advantages: (1) water-soluble carrier enabling incorporation into hydrophilic emulsions suitable for xerotic skin, whereas SLN and silicone systems typically require lipophilic bases; (2) supramolecular, non-covalent encapsulation allowing reversible retinol release, contrasted with irreversible covalent grafting in some polymer systems [22]; and (3) demonstrated stability under UV stress (90 h continuous exposure), which exceeds typical consumer exposure scenarios and was not systematically evaluated in the cited studies.

The absence of cutaneous intolerance signs observed throughout the evaluation period (0/22 participants, including 11 with self-reported sensitive skin) constitutes a noteworthy finding in terms of cutaneous tolerance. Dermocosmetic formulations containing retinol are commonly associated with subjective sensations of discomfort, including stinging, burning, or tightness, particularly in individuals with sensitive or barrier-impaired skin. The complete absence of such sensations in the present cohort, despite twice-daily application over 28 days in subjects with clinically confirmed xerosis, supports the hypothesis that PLL_G3 vectorization attenuates the cutaneous discomfort associated with retinol-containing cosmetic formulations, likely through controlled and gradual release of the active ingredient within the superficial epidermis. This is consistent with observations by Thünemann et al. [23], who reported reduced in vitro cytotoxicity of retinoic acid when complexed with poly(ethylene oxide)-b-poly(L-lysine) block copolymers, attributed to sequestration of free retinoid and pH-dependent release. However, direct comparison is limited because: (1) Thünemann’s work used retinoic acid (more irritating than retinol) and focused on in vitro models rather than clinical tolerance; (2) our study population specifically included xerotic skin (compromised barrier), whereas most retinoid tolerance studies exclude barrier-impaired participants; and (3) our twice-daily application regimen differs from the typical once-daily or alternate-day dosing used to minimize irritation in sensitive individuals.

The cutaneous tolerance observed despite twice-daily application in xerotic skin represents a noteworthy finding in the context of dermocosmetic use, though it must be interpreted cautiously given the open-label design.

Our corneometric findings (+75.8% hydration gain at 3 h for PLL_G3 formulation vs. +31.9% for control) can be compared to published moisturizer studies. Loden [42] reported hydration increases of 40–60% at 3 h for conventional glycerin-based emollients in xerotic skin (*n* = 25, corneometry), while Rawlings & Harding [33] documented 30–50% gains for ceramide-containing formulations. Our PLL_G3 formulation’s superior hydration persistence (+75.8% at 3 h) may reflect:

- Additive effects: The formulation combines humectants (glycerin 3%), emollients (sweet almond oil, isopropyl palmitate), and the PLL_G3–retinol complex. The cationic poly-L-lysine moiety may enhance electrostatic interactions with the negatively charged stratum corneum, promoting water retention [26,27].
- Retinol biological activity: After 28 days of repeated use, retinol’s effects on epidermal differentiation and barrier lipid synthesis [1,2] may contribute to improved baseline hydration capacity, though this was not directly measured in our short-term (single application) corneometry study.
- Dendrimer size effect: We previously reported [28] that larger dendrimers (G3, ~7 nm) provide greater hydration persistence than smaller dendrimers (G2, ~4.5 nm), potentially due to increased water-holding capacity in the dendritic interior (~144 nm^3^ free volume for G3, Table 8).

However, our control formulation (without retinol–PLL_G3) also showed substantial hydration gain (+31.9% at 3 h), indicating that the base emulsion contributes significantly. This contrasts with some retinoid studies where vehicle-alone controls show minimal hydration effects [10,43], likely because our formulation was specifically designed as a barrier-oriented emulsion for xerosis rather than a conventional retinoid vehicle.

The improvements in skin dryness (−43.8%), damaged appearance (−48.4%), and suppleness (+89.0%) after 28 days are consistent with meta-analyses of emollient therapy in xerosis. Proksch et al. [39] reported mean improvements of 35–50% in functional dryness scores with intensive moisturization over 4 weeks. Our results fall within or slightly exceed this range, though the absence of a vehicle control in our 28-day study limits definitive attribution of effects specifically to retinol or PLL_G3 vectorization versus the overall formulation.

Published retinoid studies in photoaging typically report longer timelines (12–24 weeks) for visible improvement [5,6,7,8,9], reflecting the time required for epidermal remodeling. Our 28-day endpoint was selected based on xerosis treatment timelines (where barrier improvement occurs within 2–4 weeks) rather than photoaging models, limiting comparability with long-term retinoid efficacy studies.

Overall, these results support a dual mechanism of action combining controlled delivery of retinol with enhanced hydration and mechanical properties of the stratum corneum. The integration of PLL_G3 dendritic vectorization into a barrier-oriented formulation design thus represents a next-generation strategy for reconciling the efficacy of retinoids with the preservation of the epidermal barrier, enabling safer and more effective repeated dermatological applications.

While the present 28-day functional evaluation provides encouraging evidence of the performance of the PLL_G3-vectorised retinol system, several aspects should be considered to guide future investigations. The exploratory design, conducted in an open-label setting, was chosen to rapidly assess feasibility, tolerance and preliminary efficacy. As with any non-comparator study, the absence of a parallel vehicle or active control limits direct quantitative comparison with conventional retinoid formulations. Nevertheless, the complementary short-term hydration experiment partly addresses this point by demonstrating the specific contribution of the retinol–PLL_G3 complex to hydration persistence.

The study population size (n = 22) was appropriate for a proof-of-concept approach aimed at detecting functionally relevant trends, although larger cohorts will be required to enable subgroup analyses and to further refine statistical robustness. Similarly, the 28-day follow-up period was well suited to assess early efficacy and tolerance, but extended studies would be valuable to explore long-term benefits, chronic use tolerance and photoageing outcomes.

The demographic profile of the volunteers, predominantly elderly and female, reflects the target population for xerosis management; however, broader inclusion in future trials will allow evaluation across different ages, genders and skin types. Finally, only one concentration of vectorised retinol was investigated, and future dose–response studies may help optimise the formulation further.

Overall, these considerations do not detract from the consistency of the observed effects, but rather define clear perspectives for larger, comparative dermocosmetic studies, including controlled intra-individual designs and extended use panels, to further substantiate the functional benefits and cutaneous tolerance profile of the PLL_G3 delivery platform in target consumer populations.

## 5. Conclusions

This study demonstrates that third-generation poly-L-lysine dendrimers provide a functionally relevant strategy for improving topical retinol delivery. By enabling controlled, non-irritating release and enhanced stability, PLL_G3-based vectorization overcomes key limitations of conventional retinoids, including intolerance and barrier disruption. The favorable tolerance profile observed after repeated use, together with sustained hydrating and reparative effects on xerotic skin, supports the suitability of this approach for sensitive, barrier-impaired skin. Overall, PLL_G3-mediated retinol delivery emerges as a promising next-generation approach for improving the cutaneous tolerance and functional efficacy of topical retinol-containing formulations in dermocosmetic practice.

## Figures and Tables

**Table 1:** Baseline demographic and functional characteristics of study.

Characteristic	Value
Total enrolled (*n*)	22
Completed study (*n*, %)	22 (100%)
Sex	
Female, *n* (%)	19 (86.4%)
Male, *n* (%)	3 (13.6%)
Age	
Mean ± SD (years)	63 ± 8.6
Range (years)	34–70
Median (years)	66.5
Ethnicity/Race	
Caucasian, *n* (%)	20 (90.9%)
North African, *n* (%)	2 (9.1%)
Fitzpatrick Skin Type	
Type II, *n* (%)	8 (36.4%)
Type III, *n* (%)	12 (54.5%)
Type IV, *n* (%)	2 (9.1%)
Baseline ODS Score	
Mean ± SD	3.2 ± 0.6
Range	2–4
Self-reported sensitive skin, *n* (%)	11 (50%)

**Table 2:** (**a**) HPLC parameters for retinol and PLL_G3 analysis. (**b**) HPLC elution programme for PLL_G3 analysis.

**(a)**			
**Parameter**	**Retinol Analysis**	**Poly-L-Lysine Analysis**	
**Instrumentation**			
Pump Module	Waters Alliance e2695	Waters Alliance e2695	
Detector	Waters 2998 Photodiode Array Detector	Waters 2998 Photodiode Array Detector	
Column	Waters XBridge Peptide BEH C18, 300 Å, 3.5 μm, 4.6 × 250 mm	Waters XBridge Peptide BEH C18, 300 Å, 3.5 μm, 4.6 × 250 mm	
**Mobile Phase**			
Eluent A	H2O + 0.1% trifluoroacetic acid	H2O + 0.1% trifluoroacetic acid	
Eluent B	Acetonitrile + 0.1% TFA	Acetonitrile + 0.1% TFA	
**Analytical Method Conditions**			
Mode	Isocratic	Gradient Linear	
Flow Rate	0.80 mL/min	0.80 mL/min	
Elution Program	Eluent A: 30%/Eluent B: 70%	Gradient: Table 2	
Detector Range	210–400 nm (resolution: 1.2 nm)	210–400 nm (resolution: 1.2 nm)	
Chromatogram Wavelength	326 nm	210 nm	
Column Temperature	Ambient temperature	Ambient temperature	
Acquisition Time	10 min	25 min	
Column Re-equilibration Time	15 min	15 min	
**(b)**			
**Time (min)**	**Flow Rate (mL/min)**	**% Eluent A**	**% Eluent B**
0	0.8	95	5
2	0.8	90	10
20	0.8	50	50
21	0.8	0	100
25	0.8	0	100

**Table 3:** Evaluation of stability Criteria [18,19,20,21].

Evaluation Criteria	Criteria	Assessment
Appearance	The product’s physical form (gel, cream, etc.) should remain consistent throughout the testing period.	Visual inspection at designated intervals (Day 0, Day 15, 1 month, 3 months).
Color	The color should remain within acceptable limits, without significant yellowing or discoloration.	Visual comparison against the reference sample stored at 25 °C in the dark. (Day 0, Day 15, 1 month, 3 months).
pH Level	The pH should remain within the specified range (e.g., 5.5 ± 0.5) to ensure product stability and skin compatibility.	pH measurement using a calibrated pH meter at designated intervals. (Day 0, Day 15, 1 month, 3 months).
Consistency and Viscosity	The consistency and viscosity should remain stable to ensure the product’s application properties are maintained.	Rheological measurements using a viscometer or rheometer at designated intervals. (Day 0, Day 15, 1 month, 3 months).
Overall Stability	The product should exhibit stability under accelerated aging conditions (45 °C), extreme cold (4 °C), and UV light exposure.	Evaluation of appearance, color, and pH stability under these conditions to determine any changes.

**Table 4:** Stability test conditions.

Conditions of Test	Descriptions of Test	Duration
Reference conditions	This product serves as a reference for all other test samples. It is stored at 25 °C and away from the light.	3 months
Accelerated aging conditions	Incubator at 45 °C in the darkness.	3 months
Extreme conditions	Fridge at 4 °C and in the darkness.	1 month
Extreme conditions	Incubator UV light (45 °C).	90 h of radiation (or 7 working days)

**Table 5:** Inclusion and non-inclusion criteria.

**INCLUSION CRITERIA** - Healthy male or female volunteers - Age between 18 and 70 years - Clinically assessed dry to very dry facial skin, defined as Overall Dry Skin (ODS) score ≥ 2 (on a 0–4 scale), as evaluated by a dermatologist at screening visit - Normal clinical examination prior to inclusion - No history of severe allergies and no active atopic flare at inclusion - No dermatological lesions on the investigated site(s), other than xerosis - No significant history of allergic reactions to cosmetic or household products - Prior experience with moisturizing skincare products (participants must have previously used facial moisturizers or body lotions within the past 12 months to ensure familiarity with topical application and to minimize novelty effects). - No specific requirement regarding retinoid exposure; both retinoid-naive and retinoid-experienced participants were eligible. - Prior retinoid use, if any, was documented but did not constitute an inclusion or exclusion criterion. - Written, free, informed, and explicit consent obtained - Ability to understand and comply with study requirements - For female participants: non-pregnant, not breastfeeding, and not planning pregnancy during the study
**NON-INCLUSION CRITERIA** - Presence of any general medical condition incomlimitapatible with study participation - Presence of an active or progressive dermatological disease - Current treatment with anti-inflammatory drugs, corticosteroids, antihistamines, or any medication likely to reduce or inhibit inflammatory or allergic reactions - Participation in another clinical study during the exclusion period

**Table 6:** Results of stability tests.

Test Condition	Parameter	Baseline (T0)	15 Days	1 Month	3 Months	Acceptance Criteria	Assessment
45 °C (accelerated)	Appearance	Homogeneous gel-cream	Homogeneous gel-cream	Homogeneous gel-cream	Homogeneous gel-cream	Physical form unchanged (no phase separation)	Conform
45 °C (accelerated)	Color	White	White	White	Off-white	No significant discoloration vs. 25 °C reference	Conform (minor acceptable change)
45 °C (accelerated)	pH	5.50 ± 0.15	5.48 ± 0.22	5.46 ± 0.25	5.43 ± 0.15	5.5 ± 0.5	Conform
45 °C (accelerated)	Viscosity (cP)	40,000 ± 12,000 (M5V2.5)	38,500 ± 2100	37,200 ± 2400	35,800 ± 2800	>10,000	Conform
4 °C (cold)	pH	5.50 ± 0.15	5.50 ± 0.20	5.50 ± 0.17	5.47 ± 0.23	5.5 ± 0.5	Conform
25 °C (reference)	pH	5.50 ± 0.15	5.57 ± 0.25	5.60 ± 0.30	5.73 ± 0.15	5.5 ± 0.5	Conform
UV stress	Color	White	Slight yellowing	–	–	Minimal change vs. reference	Conform (stress condition)
UV stress	pH	5.50 ± 0.05	5.43 ± 0.06	–	–	5.5 ± 0.5	Conform

**Table 7:** Cosmetic performance assessed by expert and volunteers after 28 days of repeated use (*n* = 22).

Parameter	Evaluator	Baseline (J1)	Baseline Range	Day 28 (J28)	Day 28 Range	Change (%)	Statistical Test	*p*-Value
Damaged skin	Dermatology expert	7.2 ± 0.9	6–9	3.7 ± 0.8	2–5	−48.4%	Wilcoxon signed-rank	<0.0001
Skin dryness	Dermatology expert	7.7 ± 1.1	5–9	4.3 ± 1.6	2–7	−43.8%	Wilcoxon signed-rank	<0.0001
Skin suppleness	Dermatology expert	3.3 ± 0.9	2–5	6.3 ± 1.1	4–8	+89.0%	Wilcoxon signed-rank	<0.0001
Skin tightness	Volunteers (self-assessment)	7.3 ± 1.2	5–9	2.8 ± 1.2	1–5	−62.1%	Wilcoxon signed-rank	<0.0001

Scoring scales. Damaged skin/Skin dryness/Skin suppleness: 0 = none; 10 = extreme. Skin tightness: 0 = none; 10 = very severe. Values are expressed as mean ± standard deviation.

**Table 8:** Theoretical estimation of the internal free volume and retinol-loading capacity of PLL_G3 dendrimers.

Parameter	Value/Assumption	Rationale/Notes	Reference
DGL3 diameter	7 nm	Experimental data for 22-kDa poly-L-lysine dendrimers	[26,27]
Radius (r)	3.5 nm	Half of diameter	Calculated
Total dendrimer volume (V = 4/3 π r^3^)	~180 nm^3^	Approximated spherical geometry	[41]
Percentage of free internal volume	80%	Typical for dendritic structures of this generation	[26]
Free internal volume	~144 nm^3^	0.8 × 180 nm^3^	Calculated
Estimated volume of 1 retinol molecule	~0.5 nm^3^	Based on molecular size and density (~0.95 g/cm^3^)	[41]
Theoretical max. retinol molecules per DGL3	~300 molecules	144 ÷ 0.5	Calculated
Experimental loading (this study)	77 molecules per dendrimer	Determined from encapsulation ratio (3.33 × 10^6^ IU/g)	This study
Retinol per lysine residue	~0.45	Equivalent to 1 retinol per 2 lysines	Calculated
Plausibility vs. literature	Consistent	Dendrimers often load 50–200 hydrophobic molecules depending on generation	[26,27]

## Data Availability

The data presented in this study are available from the corresponding author upon reasonable request and subject to institutional and ethical restrictions.

## Supplementary Materials

The following supporting information can be downloaded at: https://www.mdpi.com/article/10.3390/jcm15124435/s1; Table S1: Individual Clinical Assessments by Dermatology Expert (n = 22); Table S2: Individual Self-Assessment of Skin Tightness by Volunteers (n = 22).
