# Supplementary material for: An Innovative Dendrimer-Based Retinol Delivery System for Xerosis Care: Stability, Tolerance, and Sustained Hydration
Source: J Clin Med. 2026 Jun 8;15(12):4435. doi: 10.3390/jcm15124435 (PMC13302659; doi:10.3390/jcm15124435)
Supplement: Supplementary file 1 [file jcm-15-04435-s001.zip › jcm-4123637-supplementary.pdf]

SUPPLEMENTARY MATERIAL

Table S1. Individual Clinical Assessments by Dermatology Expert (n = 22)

Repeated use over 28 days in subjects with xerotic skin. Scores range from 0 (none) to 10 (extreme). Values are reported per volunteer, with mean ± SD in the last row.

Volunteer ID	Damaged Skin J1	Damaged Skin J28	Skin Dryness J1	Skin Dryness J28	Skin Suppleness J1	Skin Suppleness J28
**#1**	6	2	6	2	5	8
**#2**	8	5	8	5	4	7
**#3**	7	5	7	4	4	6
**#4**	7	3	8	5	3	6
**#5**	8	4	9	5	2	6
**#6**	6	3	6	3	5	8
**#7**	8	5	9	7	3	5
**#8**	7	4	7	3	3	6
**#9**	7	4	7	3	4	7
**#10**	9	4	9	6	2	5
**#11**	8	5	8	5	3	6
**#12**	9	4	9	6	2	6
**#13**	8	4	9	6	2	4
**#14**	6	3	6	3	4	7
**#15**	6	3	7	2	4	7
**#16**	7	3	7	2	3	7

**#17**	7	3	7	3	4	8
**#18**	7	4	8	6	3	5
**#19**	6	3	7	4	4	6
**#20**	7	3	7	3	4	7
**#21**	8	4	9	6	2	5
**#22**	7	4	9	6	3	6
**Mean ± SD**	7.2 ± 0.9	3.7 ± 0.8	7.7 ± 1.1	4.3 ± 1.6	3.3 ± 0.9	6.3 ± 1.1

Table S2. Individual Self-Assessment of Skin Tightness by Volunteers (n = 22)

Repeated use over 28 days. Skin tightness scored from 0 (none) to 10 (very severe).

**Volunteer ID**	**Skin Tightness J1**	**Skin Tightness J28**
**#1**	7	3
**#2**	9	3
**#3**	7	5
**#4**	7	2
**#5**	9	2
**#6**	5	2
**#7**	7	4
**#8**	6	3
**#9**	7	2
**#10**	6	3

**#11**	8	3
**#12**	9	4
**#13**	9	4
**#14**	6	4
**#15**	6	0
**#16**	9	2
**#17**	7	1
**#18**	6	1
**#19**	8	4
**#20**	7	3
**#21**	8	3
**#22**	8	3
**Mean ± SD**	7.3 ± 1.2	2.8 ± 1.2
